# Impact of advanced liver fibrosis on atrial fibrillation recurrence after ablation in non-alcoholic fatty liver disease patients

**DOI:** 10.3389/fcvm.2022.960259

**Published:** 2022-10-06

**Authors:** Zhe Wang, Yijia Wang, Fangyuan Luo, Yafei Zhai, Jiaju Li, Yinong Chen, Qing Li, Longyang Zhu, Siqi Jiao, Peng Liu, Yifeng Zhou, Yingwei Chen, Jianzeng Dong, Yihong Sun

**Affiliations:** ^1^Department of Cardiology, China-Japan Friendship Hospital, Chinese Academy of Medical Sciences & Peking Union Medical College, Beijing, China; ^2^Department of Cardiology, Beijing Hospital, Chinese Academy of Medical Sciences & Peking Union Medical College, Beijing, China; ^3^Department of Cardiology, The First Affiliated Hospital of Zhengzhou University, Zhengzhou, China; ^4^Department of Cardiology, Peking University China-Japan Friendship School of Clinical Medicine, Beijing, China; ^5^Department of Cardiology, China-Japan Friendship Hospital, Beijing, China; ^6^Department of Cardiology, Anzhen Hospital Affiliated to Capital Medical University, Beijing, China

**Keywords:** atrial fibrillation, non-alcoholic fatty liver disease, liver fibrosis, radiofrequency ablation, recurrence

## Abstract

**Aim:**

Advanced liver fibrosis is independently associated with new onset of atrial fibrillation (AF). Non-invasive liver fibrosis scores are considered an effective strategy for assessing liver fibrosis. This study aimed to investigate the association between advanced liver fibrosis and AF recurrence after ablation in patients with non-alcoholic fatty liver disease (NAFLD).

**Materials and methods:**

A total of 345 AF patients with NAFLD who underwent *de novo* ablation between 2019 and 2020 at two large hospitals in China were included in this study. AF recurrence was defined as the occurrence of atrial arrhythmia for more than 30 s by electrocardiogram or 24 h Holter monitoring after the first 3 months of ablation. Predictive values of non-alcoholic fatty liver disease fibrosis score (NFS) and Fibrosis-4 (FIB-4) scores for AF burden and recurrence after ablation were assessed.

**Results:**

At the 1 year follow-up after ablation, 38.8% of patients showed recurrence. Patients with recurrence who had higher FIB-4 and NFS scores were more likely to have persistent AF and a duration of AF ≥ 3 years. In Kaplan–Meier analysis, patients with intermediate and high NFS and FIB-4 risk categories had a higher risk of AF recurrence. Compared to patients with the low risk, intermediate and high NFS, and FIB-4 risk were independently associated with AF recurrence in multivariate Cox regression analysis (high risk: NFS, hazard ratio (HR): 3.11, 95% confidence interval (CI): 1.68∼5.76, *p* < 0.001; FIB-4, HR: 3.91, 95% CI: 2.19∼6.98, *p* < 0.001; intermediate risk: NFS, HR: 1.85, 95% CI: 1.10∼3.10, *p* = 0.020; FIB-4, HR: 2.08, 95% CI: 1.27∼3.41, *p* = 0.003).

**Conclusion:**

NFS and FIB-4 scores for advanced liver fibrosis are associated with AF burden. Advanced liver fibrosis is independently associated with AF recurrence following ablation. Advanced liver fibrosis might be meaningful in risk classification for patients after AF ablation.

## Introduction

Non-alcoholic fatty liver disease (NAFLD) has rapidly become the most common chronic liver disease, with a global prevalence of approximately 25% in adults (1). NAFLD is a multisystem disease affecting extrahepatic organs. NAFLD increases the risk of type 2 diabetes mellitus, cardiovascular disease, and chronic kidney disease. NAFLD is associated with an increased risk of cardiovascular disease/events (2), and most deaths among NAFLD patients are attributable to cardiovascular events (3). NAFLD is associated with about 2-fold greater incidence of atrial fibrillation (AF) among general individuals and about 6-fold greater incidence among populations with diabetes (4). NAFLD is also associated with an increased risk of AF in middle-aged and elderly subjects (5). The fatty liver index for advanced liver fibrosis shows a clear linear association between NAFLD and the risk of AF. NAFLD suggests a 2.1-fold risk of AF diagnosis, independent of other risk factors (6). Some risk factors linking AF and NAFLD have been shared, including insulin resistance, metabolic disorder, and inflammation (2).

Atrial fibrillation is the most common arrhythmia that has been increasing dramatically in the world. Catheter ablation is an effective approach for maintaining sinus rhythm, alleviating symptoms, and improving cardiovascular outcomes. Approximately 50% of patients develop recurrent arrhythmia within 5 years of ablation (7, 8). NAFLD is an independent risk factor for arrhythmia recurrence after AF ablation (9). Advanced liver fibrosis is associated with the incidence of AF in patients with NAFLD (10). To date, the association between liver fibrosis level and AF recurrence after radiofrequency catheter ablation (RFCA) has not been identified. This study aimed to investigate the relationship between AF recurrence after RFCA and advanced liver fibrosis, as determined using two non-invasive scoring systems in AF patients with NAFLD.

## Materials and methods

### Study design and populations

We conducted a retrospective study of AF patients admitted for RFCA from January 2019 to December 2020 to the First Affiliated Hospital of Zhengzhou University and China-Japan Friendship Hospital. The inclusion criteria were as follows: ➀ admitted to the hospital for the first RFCA treatment; ➁ diagnosed with NAFLD. The exclusion criteria were hypertrophic cardiomyopathy, advanced valvular heart disease, end-stage renal disease, and thyroid dysfunction. Patients who died or lost to follow-up were also excluded from the analysis. As the diagnostic accuracy of advanced fibrosis using the NAFLD fibrosis score (NFS) and Fibrosis-4 (FIB-4) was low in patients under 35 years, we excluded these patients (11).

The study protocol adhered to the principles of the Declaration of Helsinki and was approved by the local ethical review board. All enrolled patients provided written informed consent to participate in this study.

### Clinical and laboratory data

The following data were collected for all patients: demographic parameters, comorbidities, echocardiography parameters, and medications on admission. Echocardiography parameters included the left ventricular end-diastolic diameter, left ventricular ejection fraction, E/A ratio, and left atrial diameter (LAD). We also collected the duration from AF diagnosis to ablation, type of AF, use of anti-arrhythmic drugs (AADs) before ablation, and use of anticoagulants within 3 months of ablation. Paroxysmal AF was defined as AF lasting for less than 7 days, and persistent AF was defined as AF lasting for more than 7 days. The duration of AF was calculated by the time from the date of initial symptom onset or first diagnosis of AF to the RFCA index date. The CHA2DS2-VASc score was calculated for each patient (12).

### Diagnosis of non-alcoholic fatty liver disease and degree of liver fibrosis

The diagnosis of NAFLD was based on the following three criteria: non-excessive alcohol consumption, detection of hepatic steatosis by ultrasound, and appropriate exclusion of other liver diseases. In this study, any degree of liver fibrosis was classified as NAFLD without secondary causes based on the Asia-Pacific Working Group on NAFLD guidelines (11). A diagnosis of fatty liver was based on ultrasonography using a 3.5 MHz transducer (Philips, Cambridge MA, USA) before ablation. Ultrasonography was performed by two experienced radiologists who were unaware of the laboratory findings. A participant was considered to have excessive alcohol consumption if it is >140 g/week for males and >70 g/week for females (13).

We calculated two non-invasive liver fibrosis scores for each participant based on the parameters collected before ablation. The liver fibrosis score was calculated by the following formula: FIB-4 = aspartate transaminase (AST, IU/L) × age (years)/[alanine aminotransferase (ALT, IU/L)^1/2^ × platelet (× 10^9^/L)], with cutoffs of 1.30 and 2.67 for low-, intermediate-, and high-risk categories, respectively. The cutoff value of advanced liver fibrosis at 2.67 was used as determined in the current study (14–16). NFS = 0.094 × body mass index (BMI, kg/m^2^) + 0.037 × age (years) + 0.99 × [AST (IU/L)/ALT (IU/L)] + 1.13 × hyperglycemia/diabetes (yes = 1, no = 0) − 0.66 × albumin (ALB, g/dL) − 0.013 × platelet count (× 10^9^/L) − 1.675, with two cutoffs at − 1.455 and 0.676 for low-, intermediate-, and high-risk categories; the cutoff value of NFS was 0.676, which was defined as advanced liver fibrosis (14, 17, 18).

### Ablation protocol

For all enrolled patients, non-vitamin K antagonist oral anticoagulant (NOAC) or warfarin with a target international normalized ratio between 2.0 and 3.0 was administered to the patients. The cardiac computed tomography angiography and transesophageal echocardiography before ablation were performed to rule out the possibility of an actual thrombus.

The RFCA procedure has been described previously (19, 20). In brief, the three-dimensional electroanatomic mapping system (CARTO, Johnson & Johnson Medical, Biosense Webster, Inc., Irvine, CA, USA) was used. Circumferential pulmonary vein isolation was performed for all patients. Linear ablation (including tricuspid isthmus line, mitral valve isthmus line, left atrial roof line) or an additional complex fractionated atrial electrogram-guided procedure were performed in selected patients, especially with persistent AF. Isolation of superior vena cava (SVC) was performed if induced tachycardia from SVC or the potential of SVC was active. If AF could not be terminated after ablation, sinus rhythm was restored by cardioversion. At the end of the procedure, circumferential pulmonary vein isolation and bidirectional block of the lines were verified, and if necessary, an additional touch-up operation was conducted.

### Outcomes and follow-up at 1 year

Recurrent AF was defined as any atrial arrhythmia lasting for more than 30 s based on a 12-lead electrocardiogram or 24 h Holter monitoring after the 3 month blanking period during 1 year of follow-up. NOACs were reinitiated after ablation and continued for at least 3 months after ablation, and non-recurrent patients might not have used anticoagulants after 3 months of ablation. All patients were prescribed AADs for 3 months after ablation to prevent an early recurrence. Subsequent use of AADs was determined by the physicians and patients (16). Patients were scheduled for follow-up in the outpatient setting at 3 month intervals during the first year after RFCA. Patients who had any symptoms related to AF were asked to immediately complete an additional outpatient visit.

### Statistical analysis

Continuous variables were compared among groups using the Student’s *t*-test or the Mann-Whitney test depending on whether the data were normally distributed. The data are described as the mean ± standard deviation (SD) or the median (Q1–Q3 quartiles). Categorical variables were compared between two groups by the χ^2^ test, and the results are presented as numbers (percentage). Multivariate Cox regression analyses were performed to investigate risk factors for AF recurrence. The hazard ratio (HR) is provided with a 95% confidence interval (CI). Variables with *p* < 0.10 in univariate analysis for AF recurrence were retained for multivariate Cox regression, including age, hypertension, persistent AF, duration of AF (≥3 years), LAD, BMI, CHA2DS2-VASc score, NFS, and FIB-4 risk categories for advanced liver fibrosis. However, variables in the score formula for advanced liver fibrosis were not together in the multivariate Cox analysis models, including age and FIB-4 or BMI and NFS. Model 1 included hypertension, duration of AF (≥3 years), persistent AF, LAD, CHA2DS2-VASc score, BMI, and FIB-4 risk categories; Model 2 included hypertension, duration of AF (≥3 years), persistent AF, LAD, CHA2DS2-VASc score, age, and NFS risk categories. The Kaplan–Meier method was used to analyze the AF-free survival rate after RFCA among groups and the log-rank test to assess statistical significance.

Further univariate analyses stratified by AF type were performed to identify the association between advanced liver fibrosis and recurrence of AF after ablation. Different models were used to evaluate the ability to predict AF recurrence. The predictive model of traditional risk was established by variables with *p* < 0.10 in univariate analysis except for NFS, and FIB-4 for AF recurrence, incorporated age, hypertension, persistent AF, duration of AF (≥3 years), LAD, BMI, and CHA2DS2-VASc score. For clinical models 5 and 6, we added FIB-4 risk categories and NFS risk categories to the traditional risk model, respectively. The discriminatory abilities of clinical models 5 and 6 were assessed by the reclassification performance of each using the area under the receiver operating characteristic curve (AUC), relative integrated discrimination improvement (IDI), and category-free net reclassification improvement (NRI) values. A *p*-value < 0.05 was considered statistically significant. The statistical analyses were conducted using R software (version 4.0.3) and SPSS software (version 21.0).

## Results

### Patient characteristics

A total of 1,587 patients with AF who underwent successful RFCA were screened for eligibility and 345 patients with AF were included in this analysis, as described in the flowchart (Supplementary Figure 1). The mean time from the ultrasound test to RFCA was 2.9 ± 1.2 days in all patients. The mean age was 62.1 ± 9.4 years, and 33.6% (116/345) were female. The percentage of patients with PAF was 59.7% (206/345). A total of 91.9% (317/345) of patients used NOACs and 8.1% (28/345) warfarin within 3 months of ablation. The use of NOACs was not different between those with high NFS and FIB-4 risk category, compared with low-risk category. The proportions of patients with low-, intermediate-, and high-risk FIB-4 were 29.9% (103), 55.7% (192), and 14.5% (50), respectively. According to NFS risk categories, 27.0% (93), 57.1% (197), and 15.9% (55) of the patients were classified into low, intermediate, and high-risk categories, respectively.

Overall, 38.8% (134/345) of the patients experienced a recurrence of AF during the 1 year follow-up. Compared to those without AF recurrence, patients with recurrence were older (63.4 ± 9.0 vs. 61.2 ± 9.5, *p* = 0.033), more likely to have hypertension (59.0% vs. 47.9%, *p* = 0.044) and persistent AF (52.2% vs. 32.7%, *p* < 0.001), duration of AF ≥ 3 years (50.7% vs. 34.6%, *p* = 0.003), higher CHA2DS2-VASc score [3 (2, 4) vs. 2 (1, 4), *p* = 0.021], and larger LAD (41.9 ± 6.2 mm vs. 39.3 ± 5.9 mm, *p* < 0.001). Notably, patients with recurrence had higher NFS [−0.34 (−1.22, 0.60) vs. −1.00 (−1.65, −0.05), *p* < 0.001] and FIB-4 [1.71 (1.39, 2.66) vs. 1.53 (1.19, 2.01), *p* = 0.001]. However, there were no differences in BMI, E/A ratio, SVC ablation, linear ablation, and the use of NOACs within 3 months of ablation between the two groups (*p* > 0.05), as shown in Table 1. The E/A ratio was significantly higher in the populations at intermediate and high risk for advanced liver fibrosis than in the low-risk population according to the risk stratification of FIB-4 and NFS for advanced liver fibrosis. A comparison of baseline characteristics between the three groups according to the NFS and FIB-4 risk categories is shown in Supplementary Tables 1, 2.

**TABLE 1:** Baseline characteristics of the patients with and without atrial fibrillation (AF) recurrence.

Variable	All (*n* = 345)	Non-recurrence (*n* = 211)	Recurrence (*n* = 134)	*P*-value
Age, years	62.1 ± 9.4	61.2 ± 9.5	63.4 ± 9.0	0.033
Female gender	116 (33.6)	70 (32.2)	46 (34.3)	0.825
BMI, kg/m^2^	25.9 ± 3.3	25.6 ± 3.2	26.3 ± 3.5	0.079
Current smoking	96 (27.8)	64 (30.3)	32 (23.9)	0.193
Current drinking	85 (24.6)	54 (25.6)	31 (23.1)	0.606
Hypertension	180 (52.2)	101 (47.9)	79 (59.0)	0.044
Diabetes mellitus	93 (27.0)	52 (24.6)	41 (30.6)	0.225
Dyslipidemia	200 (58.0)	121 (57.3)	79 (59.0)	0.768
CHD	104 (30.1)	62 (29.4)	42 (31.3)	0.699
Heart failure	53 (15.4)	30 (14.2)	23 (17.3)	0.460
Prior stroke	59 (17.1)	31 (14.7)	28 (20.9)	0.136
**Medication**				
ACEI/ARB	142 (41.2)	81 (38.4)	61 (45.5)	0.189
AADs of pre-ablation	233 (67.5)	139 (65.9)	94 (70.1)	0.409
Statins	135 (39.1)	76 (36.0)	59 (44.0)	0.137
**Use of anticoagulants within 3 months of ablation**				0.723
NOACs	317 (91.9)	193 (91.5)	124 (92.5)	
Warfarin	28 (8.1)	18 (8.5)	10 (7.5)	
CHA2DS2-VASc score	2 (1, 4)	2 (1, 4)	3 (2, 4)	0.021
**Laboratory test**				
TC, mmol/L	3.8 ± 1.0	3.8 ± 0.9	3.7 ± 1.0	0.566
HDL-C, mmol/L	1.1 ± 0.3	1.1 ± 03	1.1 ± 0.3	0.179
LDL-C, mmol/L	2.3 ± 0.9	2.3 ± 0.8	2.3 ± 1.0	0.879
UA, mmol/l	341.3 ± 94.6	342.1 ± 87.7	339.9 ± 105.0	0.828
Cr, μmol/l	76.1 ± 16.7	75.8 ± 17.8	76.6 ± 14.9	0.662
HS-CRP, mg/L	1.15 (0.58, 2.14)	1.10 (0.55, 2.08)	1.20 (0.63, 2.28)	0.511
AST, U/L	22 (18, 27)	21 (19, 26)	24 (18, 31)	0.276
ALT, U/L	20 (15, 28)	20 (15, 30)	20 (16, 29)	0.195
ALB, g/L	41.9 ± 4.5	42.1 ± 4.1	41.5 ± 5.2	0.236
FPG, mmol/L	5.8 ± 1.9	5.7 ± 2.0	5.8 ± 1.8	0.768
HbA1c, %	6.0 ± 1.0	6.0 ± 1.0	6.1 ± 1.1	0.246
LVEF, %	62.5 ± 8.0	62.4 ± 8.3	62.8 ± 7.5	0.602
LAD, mm	40.3 ± 6.2	39.3 ± 5.9	41.9 ± 6.2	<0.001
LVEDD, mm	48.3 ± 5.4	48.0 ± 5.6	48.6 ± 5.0	0.300
E/A ratio	0.9 ± 0.4	0.9 ± 0.4	0.9 ± 0.4	0.113
Duration of AF (≥3 years)	141 (40.9)	73 (34.6)	68 (50.7)	0.003
Type of AF				<0.001
Paroxysmal	206 (59.7)	142 (67.3)	64 (47.8)	
Persistent	139 (40.4)	69 (32.7)	70 (52.2)	
Linear ablation	149 (43.2)	85 (40.3)	64 (47.8)	0.172
SVC isolation	52 (15.1)	34 (16.1)	18 (13.4)	0.498
NFS	−0.75 (−1.52, 0.06)	−1.00 (−1.65, −0.05)	−0.34 (−1.22, 0.60)	<0.001
**NFS risk categories < 0.001**				
Low	93 (27.0)	73 (34.6)	20 (14.9)	
Intermediate	197 (57.1)	117 (55.5)	80 (59.7)	
High	55 (15.9)	21 (10.0)	34 (25.4)	
FIB-4	1.61 (1.24, 2.10)	1.53 (1.19, 2.01)	1.71 (1.39, 2.66)	0.001
FIB-4 risk categories				<0.001
Low	103 (29.9)	82 (38.9)	21 (15.7)	
Intermediate	192 (55.7)	109 (51.7)	83 (61.9)	
High	50 (14.5)	20 (9.5)	30 (22.4)	

Continuous data are presented as means ± standard deviation (SD) or median (inter-quartile range), and categorical data were shown as *n* (%). BMI, body mass index; CHD, coronary heart disease; ACEI, angiotensin-converting enzyme inhibitor; ARB, angiotensin receptor blocker; AADs, anti-arrhythmic drugs; TC, total cholesterol; HDL-C, high-density lipoprotein cholesterol; LDL-C, low-density lipoprotein cholesterol; UA, uric acid; Cr, creatinine; FPG, fasting plasma glucose; AST, aspartate transaminase; ALT, alanine aminotransferase; ALB, albumin; HS-CRP, high-sensitivity C-reactive protein; HbA1c, glycosylated hemoglobin; LAD, left atrial diameter; LVEF, left ventricular ejection fraction; LVEDD, left ventricular end-diastolic diameter; AF, atrial fibrillation; SVC, superior vena cava; NFS, non-alcoholic fatty liver disease fibrosis score; FIB-4, fibrosis-4.

### Association between fibrosis-4 and non-alcoholic fatty liver disease fibrosis score categories with a duration of atrial fibrillation ≥3 years and persistent atrial fibrillation

**FIGURE 1:**
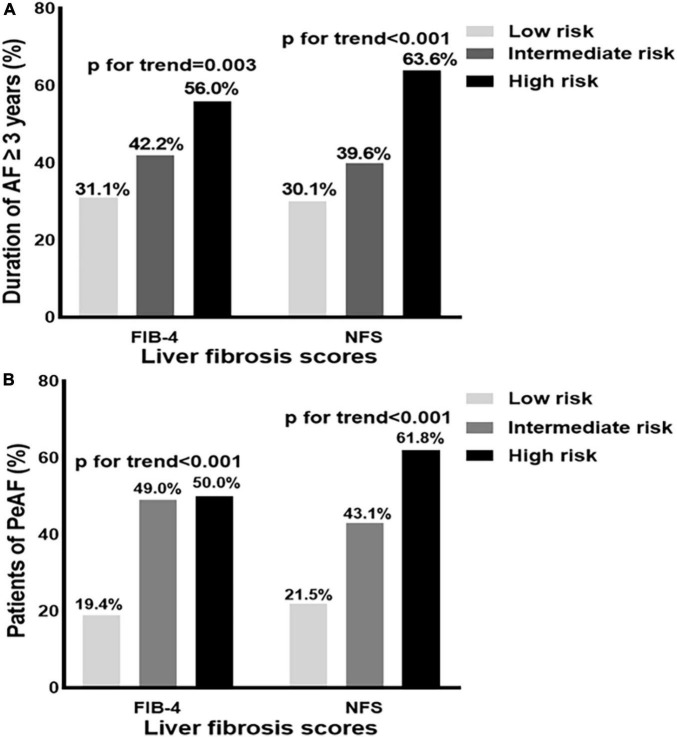
**(A,B)** Distribution of the patients with a duration of atrial fibrillation (AF) ≥ 3 years, and persistent atrial fibrillation (PeAF) according to fibrosis-4 (FIB-4), and non-alcoholic fatty liver disease fibrosis score (NFS) risk categories. AF, atrial fibrillation; PeAF, persistent atrial fibrillation; NFS, non-alcoholic fatty liver disease fibrosis score; FIB-4, fibrosis-4.

The proportion of patients with a duration of AF ≥ 3 years significantly increased with the degrees of liver fibrosis according to FIB-4 (*p* for trend = 0.003) and NFS (*p* for trend < 0.001) (Figure 1A). The proportion of patients with persistent AF was higher among those with an intermediate and high risk of FIB-4 than those with low risk (all *p* for trend < 0.001, Figure 1B).

### Association between advanced liver fibrosis and recurrence of atrial fibrillation

Recurrence rates of AF were 20.4, 43.2, and 60.0% in the low, intermediate, and high risk for advanced liver fibrosis groups, respectively, based on the FIB-4 risk categories. According to NFS risk categories, AF recurrence rates were 21.5, 40.6, and 61.8% in those with low, intermediate, and high risk, respectively (all *p* for trend < 0.001), as described in Supplementary Figure 2.

**FIGURE 2:**
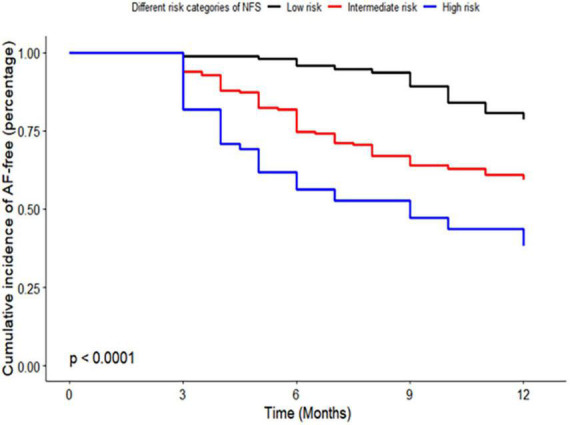
Kaplan–Meier curve of risk categories of non-alcoholic fatty liver disease fibrosis score (NFS) for atrial fibrillation (AF) recurrence in patients with non-alcoholic fatty liver disease (NAFLD). NFS, non-alcoholic fatty liver disease fibrosis score; AF, atrial fibrillation; NAFLD, non-alcoholic fatty liver disease.

**FIGURE 3:**
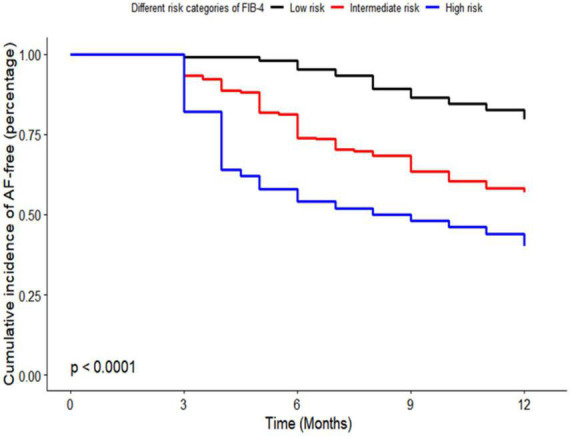
Kaplan–Meier curve of risk categories of fibrosis-4 (FIB-4) for atrial fibrillation (AF) recurrence in patients with non-alcoholic fatty liver disease (NAFLD). FIB-4, fibrosis-4; AF, atrial fibrillation; NAFLD, non-alcoholic fatty liver disease.

Kaplan–Meier curve analysis showed that patients in the intermediate and high-risk category of NFS and FIB-4 had a higher risk of recurrence than those in the low-risk category (*p* < 0.001), as depicted in Figures 2, 3.

**FIGURE 4:**
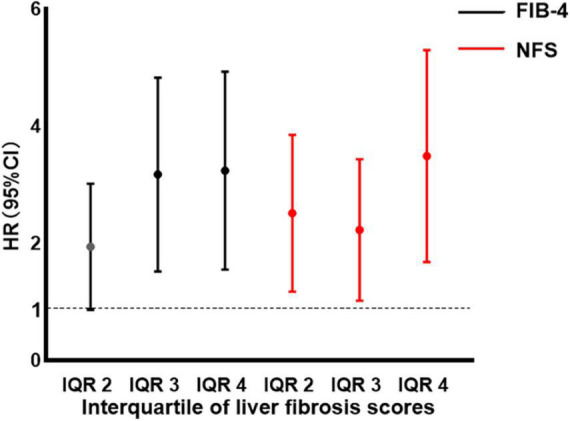
Hazard ratio (HR) by interquartile (IQR) of fibrosis-4 (FIB-4) and non-alcoholic fatty liver disease fibrosis score (NFS). Filled circles and vertical lines indicate the HR and 95% confidence interval (CI) for IQR 2–4 of FIB-4 and NFS, relative to IQR 1 of FIB-4 and NFS. IQR 1 of FIB-4: FIB-4 < 1.238; IQR 2 of FIB-4: 1.238 ≤ FIB-4 < 1.614; IQR 3 of FIB-4: 1.614 ≤ FIB-4 < 2.098; IQR 4 of FIB-4: 2.098 ≤ FIB-4. IQR 1 of NFS: NFS < −1.520; IQR 2 of NFS: −1.520 ≤ NFS < −0.746; IQR 3 of NFS: −0.746 ≤ NFS < 0.058; IQR 4 of NFS: 0.058 ≤ NFS. NFS, non-alcoholic fatty liver disease fibrosis score; FIB-4, fibrosis-4; IQR, interquartile; HR hazard ratio; CI, confidence interval.

Compared to patients with the first interquartile (IQR) of FIB-4 and NFS, the HR for AF recurrence increased significantly with FIB-4 level above IQR 2, with IQR 3 (HR: 2.89, 95% CI: 1.68∼4.96, *p* < 0.001), and IQR 4 (HR = 2.95, 95% CI 1.72∼5.06, *p* < 0.001). Patients with an IQR of 2–4 for NFS also had a significantly increased NFS level above IQR 1 (all *p* < 0.05), as illustrated in Figure 4.

### Independent risk factors for atrial fibrillation recurrence

The multivariate analysis showed that LAD, duration of AF ≥ 3 years, and higher risk categories of FIB-4 and NFS were independently associated with AF recurrence at the 1 year follow-up. The risk of AF recurrence was increased in patients with intermediate and high NFS and FIB-4 categories compared with those in the low risk category (intermediate NFS category: HR = 1.85, 95% CI: 1.10∼3.10, *p* = 0.020; high NFS category: HR: 3.11, 95% CI: 1.68∼5.76, *p* < 0.001; intermediate FIB-4 category: HR: 2.08, 95% CI: 1.27∼3.41, *p* = 0.003; high FIB-4 category: HR: 3.91, 95% CI: 2.19∼6.98, *p* < 0.001; Table 2). Moreover, the association between FIB-4 and NFS as continuous variables and AF recurrence remained significant after multivariate adjustment (NFS, HR: 1.28, 95% CI: 1.07∼1.52, *p* = 0.008; FIB-4, HR: 1.24, 95% CI: 1.12∼1.38, *p* < 0.001), as shown in Table 3. Overall, the risk of AF recurrence was significantly increased for NFS and FIB-4 groups at higher risk for advanced liver fibrosis compared with those in the low-risk group, both among patients with paroxysmal and persistent AF, as shown in Supplementary Table 3.

**TABLE 2:** Risk factors for recurrence of atrial fibrillation (AF) by multivariate Cox regression analysis model.

Variable	Model 1		Model 2	
	HR (95% CI)	*P-*value	HR (95% CI)	*P-*value
Hypertension	1.38 (0.92∼2.07)	0.117	1.25 (0.84∼1.87)	0.272
Duration of AF (≥3 years)	1.46 (1.03∼2.08)	0.035	1.46 (1.02∼2.09)	0.038
Persistent AF	1.37 (0.97∼1.95)	0.077	1.39 (0.98∼1.98)	0.066
LAD	1.05 (1.02∼1.09)	0.001	1.06 (1.03∼1.09)	<0.001
CHA2DS2-VASc score	1.04 (0.94∼1.15)	0.436	1.01 (0.89∼1.15)	0.846
BMI	1.03 (0.98∼1.08)	0.294		
Age			1.00 (0.97∼1.02)	0.826
**FIB-4 risk categories**				
Low	Reference			
Intermediate	2.08 (1.27∼3.41)	0.003		
High	3.91 (2.19∼6.98)	<0.001		
**NFS risk categories**				
Low			Reference	
Intermediate			1.85 (1.10∼3.10)	0.020
High			3.11 (1.68∼5.76)	<0.001

Model 1 included hypertension, duration of AF, persistent AF, LAD, CHA2DS2-VASc score, BMI, and FIB-4 risk categories; Model 2 included hypertension, duration of AF, Persistent AF, LAD, CHA2DS2-VASc score, age, NFS risk categories. The variables in the liver fibrosis score formula were not enrolled in the multivariate Cox analysis model. AF, atrial fibrillation; LAD, left atrial diameter; BMI, body mass index; FIB-4, fibrosis-4; NFS, non-alcoholic fatty liver disease fibrosis score; HR, hazard ratio; CI, confidence interval.

**TABLE 3:** Risk factors for recurrence of atrial fibrillation (AF) by multivariate Cox regression analysis model.

Variable	Model 3		Model 4	
	HR (95% CI)	*P-*value	HR (95% CI)	*P-*value
Hypertension	1.22 (0.82∼1.81)	0.336	1.29 (0.86∼1.92)	0.219
Duration of AF (≥3 years)	1.54 (1.08∼2.18)	0.017	1.48 (1.04∼2.11)	0.032
Persistent AF	1.56 (1.10∼2.20)	0.012	1.46 (1.03∼2.07)	0.035
LAD	1.06 (1.03∼1.09)	<0.001	1.06 (1.03∼1.09)	<0.001
CHA2DS2-VASc score	1.05 (0.95∼1.17)	0.357	1.06 (0.92∼1.22)	0.443
BMI	1.03 (0.97∼1.08)	0.368		
Age			1.00 (0.98∼1.03)	0.976
FIB-4, continuous variable	1.24 (1.12∼1.38)	<0.001		
NFS, continuous variable			1.28 (1.07∼1.52)	0.008

Model 3 included hypertension, duration of AF (≥3 years), persistent AF, LAD, CHA2DS2-VASc score, BMI, and FIB-4; Model 4 included hypertension, duration of AF (≥3 years), persistent AF, LAD, CHA2DS2-VASc score, age, and NFS. The variables in the liver fibrosis score formula were not enrolled in the multivariate Cox analysis model. AF, atrial fibrillation; LAD, left atrial diameter; BMI, body mass index; FIB-4, fibrosis-4; NFS, non-alcoholic fatty liver disease fibrosis score; HR, hazard ratio; CI, confidence interval.

### Comparison of clinical models for predicting atrial fibrillation recurrence

Addition of FIB-4 and NFS for advanced liver fibrosis as categorical variables enhanced predictive ability compared with the traditional risk model (AUC, 0.729 vs. 0.689, *p* = 0.017; 0.715 vs. 0.689, *p* = 0.063, respectively). To further evaluate the discriminatory ability of the models, we considered the following categories of risk of AF recurrence: 0–30.0% low, 30.0–70.0% intermediate, and 70.0% or more high. The cutoff values used to calculate the net reclassifications were 30 and 70%. Compared to the traditional risk model, both model 5 and 6 showed higher discriminant capacity. The incremental reclassification efficacy for predicting AF recurrence was significantly improved by adding the FIB-4 risk categories (relative IDI, 0.056, *p* < 0.001; categorical NRI, 0.153, *p* = 0.003) and NFS risk categories (relative IDI, 0.032, *p* = 0.006; categorical NRI, 0.103, *p* = 0.043), as indicated in Table 4.

**TABLE 4:** Comparison of discriminant and reclassification capacities of each model for predicting atrial fibrillation (AF) recurrence.

			Relative IDI		Categorical NRI	
	AUC	*P*-value	IDI (95% CI)	*P*-value	NRI (95% CI)	*P*-value
Traditional risk model	0.689	Reference	Reference		Reference	
Clinical model 5 (traditional risk model + FIB-4 risk categories)	0.729	0.017	0.056 (0.021∼0.102)	<0.001	0.153 (0.050∼0.255)	0.003
Clinical model 6 (traditional risk model + NFS risk categories)	0.715	0.063	0.032 (0.008∼0.073)	0.006	0.103 (0.003∼0.203)	0.043

Three predictive models were constructed as follows: traditional risk model included age, hypertension, duration of AF, persistent AF, LAD, CHA2DS2-VASc score, and BMI; Clinical model 4 included a combination of traditional risk model and FIB-4 risk categories; Clinical model 5 included a combination of traditional risk model and NFS risk categories. AF, atrial fibrillation; LAD, left atrial diameter; BMI, body mass index; FIB-4, fibrosis-4; NFS, non-alcoholic fatty liver disease fibrosis score; AUC, area under the receiver operating characteristic curve; CI, confidence interval; IDI, integrated discrimination improvement; NRI, net reclassification improvement.

## Discussion

In this exploratory study, we found that the recurrence rate of AF was 38.8% in NAFLD patients after the first ablation. Higher FIB-4 and NFS risk categories for advanced liver fibrosis were observed in patients with persistent AF and duration of AF ≥ 3 years. Importantly, high-risk categories of FIB-4 and NFS were independently associated with a high risk of AF recurrence after ablation, constituting useful predictors for AF recurrence in NAFLD patients.

RFCA can improve symptoms and quality of life related to AF (8). However, the recurrence rate was 10–30% for patients with paroxysmal AF and 25–35% for patients with persistent AF during 1 year follow-up (7). NAFLD may represent a common determinant of the risk of several cardiovascular diseases. A systematic review and meta-analysis found that patients with NAFLD are at a higher risk of myocardial infarction, ischemic stroke, heart failure, and AF in NAFLD patients compared with patients without NAFLD (21). NAFLD is associated with an increased risk of persistent or permanent AF in diabetes mellitus patients (22). One cohort study reported a higher recurrence rate of AF of 56% (50/89) in NAFLD patients compared with 21% (37/178) without NAFLD during a mean follow-up of 29 months, and NAFLD is an independent risk factor for recurrence after ablation (9). Individuals with NAFLD are more likely to exhibit impaired lipid metabolism than healthy control individuals (23). In this study, we found high recurrence rates of AF of 31.1 and 50.4% in patients with paroxysmal AF and persistent AF, respectively. This may be because the patients included all patients with NAFLD in this study, suggesting that NAFLD may reduce the AF ablation effect. This result further stresses the point that NAFLD has an important influence on the recurrence of AF.

We found a lower E/A ratio for advanced hepatic fibrosis, compared with a low risk of NFS and FIB-4, which indicates that advanced hepatic fibrosis is associated with the worsening of left ventricular diastolic function. Advanced liver fibrosis is considered a hepatic manifestation of metabolic syndrome that can have deleterious effects on cardiac function (24). NFS and FIB-4 scores are well-accepted and validated markers for advanced liver fibrosis (14). In a prospective study with patients over 65 years of age, NFS and FIB-4 scores were superior to other liver fibrosis scores in predicting cardiovascular events (25). Another study found that the prevalence of persistent and permanent AF was significantly higher in proportion to those with a high FIB-4 (≥2.51), and that the FIB-4 index was an independent prognostic indicator for identifying AF type and load (26). Both NFS and FIB-4 scores are independently associated with new-onset AF in patients with NAFLD (27). Our study found a significant correlation between NFS and FIB-4 risk categories and type of AF and duration of AF, which suggests that advanced liver fibrosis is associated with AF burden. In general, the level of advanced liver fibrosis can reflect the degree of metabolic disorder, AF closely related to metabolic disorders (28). Advanced liver fibrosis in patients with NAFLD is reversible, indicating that interventions may be able to reverse advanced liver fibrosis and effectively prevent the development of AF (29).

Several risk factors for recurrence of AF after ablation have been identified, such as LAD, long duration of AF, metabolic syndrome, and insulin resistance (7). Our study also found a large LAD and duration of AF ≥ 3 years to be independent risk factors for recurrence after RFCA, consistent with previous studies (8). Importantly, we reveal that advanced liver fibrosis is an independent risk factor for AF recurrence. A previous study found that myocardial steatosis and the increase of epicardial adipose tissue may produce adverse reactions, resulting in dysfunction of myocardial function and structure and promoting arrhythmia in NAFLD patients (30). It has been reported that lifestyle changes, such as dietary changes, physical activity, and weight control, may decrease advanced liver fibrosis in patients with NAFLD (29). In AF management, adherence to the ABC pathway is recommended by the most recent guidelines on AF management and the positive impact of the pathway has already been found in some studies (31, 32). Comprehensive risk-factor modification and interventions that target underlying patient conditions have led to a reduction in AF burden and recurrence after ablation. And weight reduction and physical activity may reduce the occurrence and development of AF (33). Thus, lifestyle management may be a bridge between liver fibrosis and AF. Improving advanced liver fibrosis may have important clinical significance for the treatment of AF patients with NAFLD.

Some intertwined pathophysiological mechanistic links between advanced liver fibrosis and AF have been proposed, including inflammation, metabolic disorders, and autonomic dysfunction (10). One possible pathway involves the association between advanced hepatic fibrosis and chronic activation of proinflammatory transcription factors, which may promote cardiac fibrosis and the formation of atrial low-voltage areas and contribute to atrial arrhythmogenicity (23, 28). Patients who have advanced hepatic fibrosis often have metabolic disorders, including impaired glucose homeostasis, insulin resistance, and abnormal lipid metabolism, which may slow electrical conduction in the atrial and aggravate cardiac electrical remodeling (34, 35). Additionally, advanced liver fibrosis may be associated with an abnormal autonomic activity. On the one hand, vagus nerve stimulation may lead to a decrease in the atrial refractory period and an increase in the atrial refractory dispersion. On the other hand, abnormal sympathetic activity may cause myocardial injury and alter intracellular ion currents, leading to instability of cardiac electrical activity. The autonomic nervous system may be a potent modulator of the initiation and perpetuation of arrhythmia (35, 36). We found a significant impact of advanced liver fibrosis on the efficacy of RFCA in patients with NAFLD. Nevertheless, whether treatment for advanced liver fibrosis can reduce the burden of AF requires further research. Large, prospective studies are needed to confirm the effect of advanced liver fibrosis on AF recurrence in patients with NAFLD.

## Limitations

Some potential limitations need to be acknowledged. First, the study had a retrospective nature, and selection bias could not be avoided. We cannot completely rule out the possibility that some patients had other liver diseases with undetected hepatic steatosis. Further examination will be necessary to assess cardiac fibrosis by cardiac magnetic resonance in patients. Second, we did not use a strict monitoring device for detecting asymptomatic arrhythmia, which limits the generalizability of the results. Third, the study is limited by the potential for unmeasured confounding variables. We did not completely collect data for inflammation markers associated with AF progression. HS-CRP was available for only 65.7% of AF recurrence patients and 67.8% of recurrence patients. There was no record parameter of obstructive sleep apnea, left atrial volume index (LAVI) by echocardiography, the results of AF patient’s 3D mapping, and bleeding events in our study. We used LAD instead of LAVI, and previous studies revealed that LAD is an associated risk factor with AF development (7). Hepatic patients may have more bleeding events compared to patients without hepatic involvement. Identifying those with high bleeding risk, NAFLD patients may help make a further risk assessment. Finally, the use of ultrasound for the diagnosis of NAFLD is non-quantitative and less accurate for detecting mild hepatic steatosis. Regardless, it is inexpensive, non-invasive, and well accepted in daily practice.

## Conclusion

This study demonstrates that FIB-4 and NFS indexes for advanced liver fibrosis are associated with AF burden. Advanced liver fibrosis is independently associated with AF recurrence after RFCA in AF patients with NAFLD and suggesting a more careful evaluation and better risk-stratification for the AF patients affected by hepatic fibrosis before a planned ablation.

## Data availability statement

The raw data supporting the conclusions of this article will be made available by the authors, without undue reservation.

## Ethics statement

The studies involving human participants were reviewed and approved by the Ethics Committee of The First Affiliated Hospital of Zhengzhou University. The patients/participants provided their written informed consent to participate in this study.

## Author contributions

ZW, YW, FL, and JL designed the study and wrote the draft of the manuscript. YaZ, LZ, SJ, YNC, and QL collected the clinical data and performed data analysis. PL, YiZ, YWC, JD, and YS verified the data extraction and reviewed the manuscript. All authors performed the study and approved the final manuscript.
