# Supplementary material for: Impact of advanced liver fibrosis on atrial fibrillation recurrence after ablation in non-alcoholic fatty liver disease patients
Source: Front Cardiovasc Med. 2022 Oct 6;9:960259. doi: 10.3389/fcvm.2022.960259 (PMC9583404; doi:10.3389/fcvm.2022.960259)
Supplement: Supplementary file 1 [file Data_Sheet_1.docx]

Supplemental Figure 1. The flowchart of the study cohort. AF, atrial fibrillation; RFCA, radiofrequency catheter ablation; NAFLD, nonalcoholic fatty liver disease.


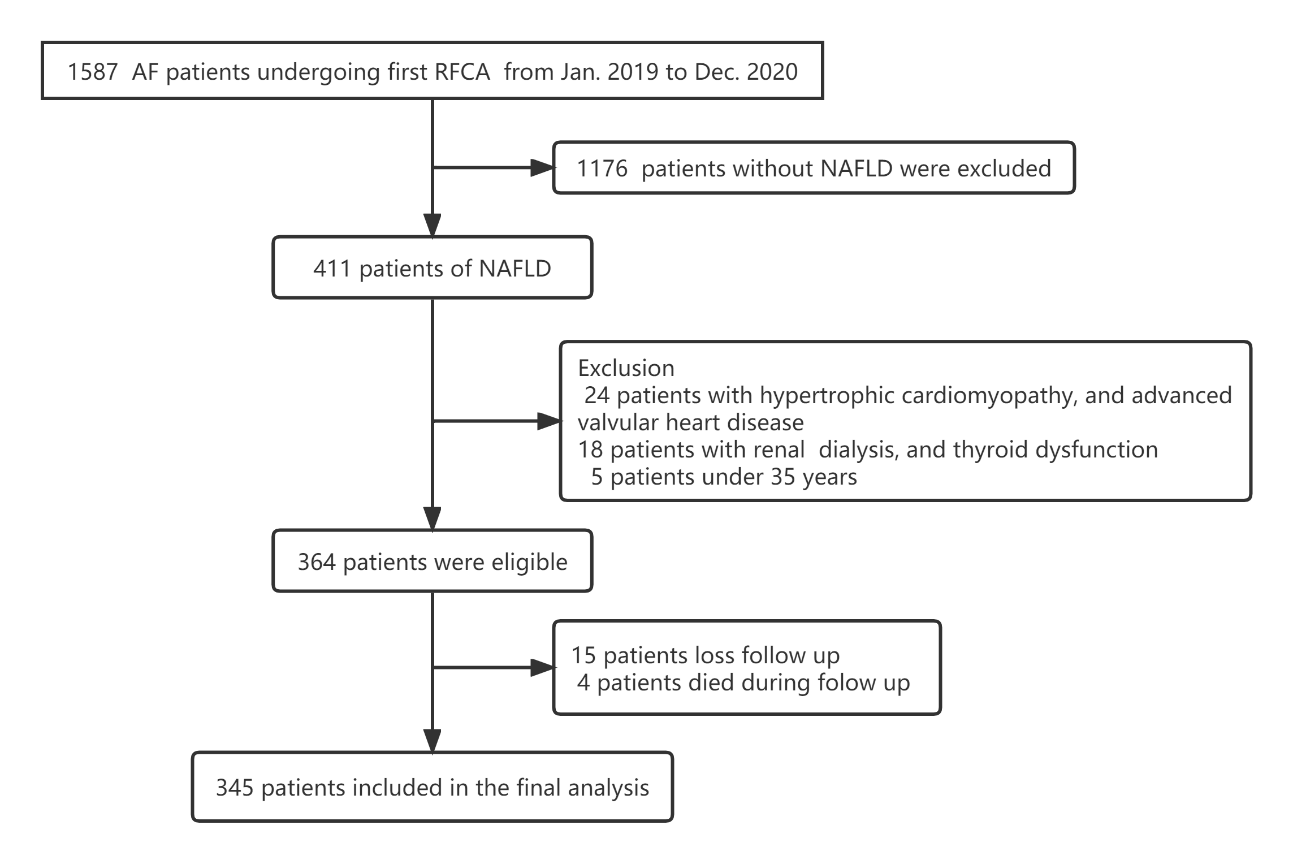


Supplemental Figure 2. Distribution of the patients with recurrence of AF according to fibrosis-4 (FIB-4) and nonalcoholic fatty liver disease fibrosis score (NFS) risk categories. AF, atrial fibrillation; NFS, nonalcoholic fatty liver disease fibrosis score; FIB-4, fibrosis-4.


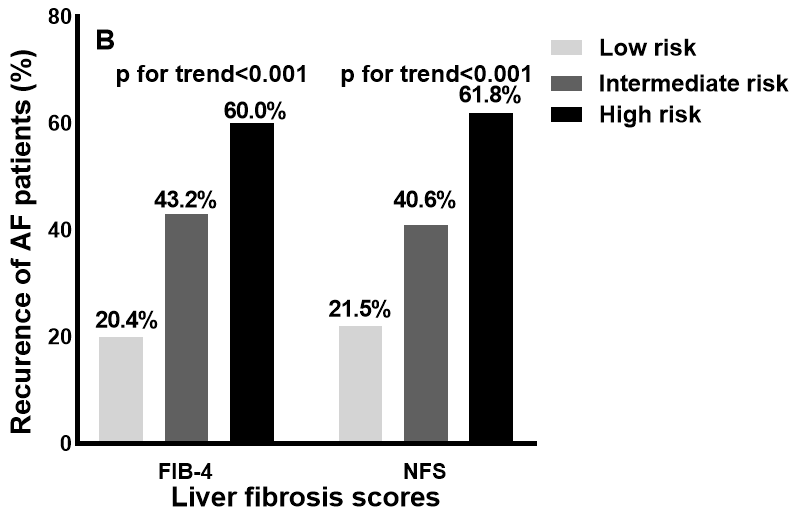


Supplemental Table 1. Comparison of baseline characteristics in atrial fibrillation (AF) patients according to nonalcoholic fatty liver disease fibrosis score (NFS) risk categories.

| Variable | Low risk | Intermediate risk | High risk | *p*-value |
| --- | --- | --- | --- | --- |
| n | 93 | 197 | 55 |  |
| Age, years | 56.0±10.4 | 63.6±7.6 | 66.6±8.5 | <0.001 |
| Female gender | 27(29.0) | 67(34.0) | 22(40.0) | 0.388 |
| BMI, kg/m^2^ | 25.2±3.1 | 25.8±3.4 | 27.4±3.1 | <0.001 |
| Current smoking | 26(28.0) | 56(28.4) | 14(25.5) | 0.909 |
| Current drinking | 27(29.0) | 44(22.3) | 14(25.5) | 0.461 |
| Hypertension | 37(39.8) | 111(56.3) | 32(58.2) | 0.019 |
| Diabetes mellitus | 21(22.6) | 50(25.4) | 22(40.0) | 0.052 |
| Dyslipidemia | 46(49.5) | 125(63.5) | 29(52.7) | 0.055 |
| Duration of AF (≥3 years) | 28(30.1） | 78(39.6) | 35(63.6) | <0.001 |
| Type of AF |  |  |  | <0.001 |
| Paroxysmal | 73(78.5) | 112(56.9) | 21(38.2) |  |
| Persistent | 20(21.5) | 85(43.1) | 34(61.8) |  |
| CHD | 25(26.9) | 65(33.0) | 14(25.5) | 0.406 |
| Heart failure | 12(12.9) | 29(14.7) | 12(21.8) | 0.323 |
| Prior stroke | 18(19.4) | 33(16.8) | 8(14.5) | 0.739 |
| Medication |  |  |  |  |
| ACEI/ARB | 30(32.3) | 86(43.7) | 26(47.3) | 0.111 |
| Pre-ablation AADs | 59(63.4) | 139(70.6) | 35(63.6) | 0.384 |
| Statins | 25(26.9) | 92(46.7) | 18(32.7) | 0.003 |
| Cr, μmol/l | 76.5±22.9 | 75.9±15.1 | 75.5±13.9 | 0.806 |
| HS-CRP, mg/L | 1.06(0.47,1.85) | 1.15(0.60,2.13) | 1.53(0.59,2.54) | 0.357 |
| LVEF, % | 63.3±6.8 | 62.0±8.8 | 63.2±6.4 | 0.338 |
| LAD, mm | 38.8±6.1 | 41.0±6.2 | 40.7±5.0 | 0.015 |
| E/A ratio | 1.0±0.4 | 0.9±0.4 | 0.7±0.3 | <0.001 |

Note: Continuous data are presented as means±standard deviation (SD) or median (inter-quartile range), and categorical data were shown as n (%).

Abbreviations: AF, atrial fibrillation; BMI, body mass index; CHD, coronary heart disease; ACEI, angiotensin-converting enzyme inhibitor; ARB, angiotensin receptor blocker; AADs, anti-arrhythmic drugs; Cr, creatinine; HS-CRP, high-sensitivity C-reactive protein; LAD, left atrial diameter; LVEF, left ventricular ejection fraction; NFS, nonalcoholic fatty liver disease fibrosis score.

Supplemental Table 2. Comparison of baseline characteristics in atrial fibrillation (AF) patients according to fibrosis-4 (FIB-4) risk categories.

| Variable | Low risk | Intermediate risk | High risk | *p*-value |
| --- | --- | --- | --- | --- |
| n | 103 | 192 | 50 |  |
| Age, years | 57.0±10.0 | 64.2±8.2 | 64.3±8.1 | <0.001 |
| Female gender | 31(30.1%) | 70(36.5) | 15(30.0) | 0.459 |
| BMI, kg/m^2^ | 25.2±3.1 | 25.8±3.4 | 27.4±3.1 | 0.889 |
| Current smoking | 32(31.1) | 52(27.1) | 12(24.0) | 0.620 |
| Current drinking | 31(30.1) | 46(24.0) | 8(16.0) | 0.156 |
| Hypertension | 52(50.5) | 107(55.7) | 21(42.0) | 0.206 |
| Diabetes mellitus | 35(34.0) | 48(25.0) | 10(20.0) | 0.123 |
| Dyslipidemia | 61(59.2) | 117(60.9) | 22(44.0) | 0.092 |
| Duration of AF (≥3 years) | 32(31.1） | 81(42.2) | 28(56.0) | 0.011 |
| Type of AF |  |  |  | <0.001 |
| Paroxysmal | 83(80.6) | 98(51.0) | 25(50.0) |  |
| Persistent | 20(19.4) | 94(49.0) | 25(50.0) |  |
| CHD | 32(31.1) | 61(31.8) | 11(22.0) | 0.395 |
| Heart failure | 12(11.7) | 36(18.8) | 5(10.0) | 0.143 |
| Prior stroke | 16(15.5) | 37(19.3) | 6(12.0) | 0.420 |
| Medication |  |  |  |  |
| ACEI/ARB | 38(36.9) | 84(43.8) | 20(40.0) | 0.513 |
| Pre-ablation AADs | 67(65.0) | 131(68.2) | 35(70.0) | 0.790 |
| Statins | 25(26.9) | 92(46.7) | 18(32.7) | 0.028 |
| Cr, μmol/l | 76.4±19.4 | 75.9±15.9 | 76.2±14.1 | 0.967 |
| HS-CRP, mg/L | 1.01(0.53,1.84) | 1.11(0.58,2.10) | 1.80(0.69,3.15) | 0.052 |
| LVEF, % | 63.8±6.7 | 62.3±8.1 | 60.8±9.5 | 0.070 |
| LAD, mm | 38.8±6.1 | 41.0±6.2 | 40.7±5.0 | 0.041 |
| E/A ratio | 1.0±0.4 | 0.9±0.4 | 0.7±0.3 | 0.002 |

Note: Continuous data are presented as means±standard deviation (SD) or median (inter-quartile range), and categorical data were shown as n (%).

Abbreviations: AF, atrial fibrillation; BMI, body mass index; CHD, coronary heart disease; ACEI, angiotensin-converting enzyme inhibitor; ARB, angiotensin receptor blocker; AADs, anti-arrhythmic drugs; Cr, creatinine; HS-CRP, high-sensitivity C-reactive protein; LAD, left atrial diameter; LVEF, left ventricular ejection fraction; FIB-4, fibrosis-4.

Supplemental Table 3. The incidence of atrial fibrillation (AF) recurrence stratified by the type of AF.

| Variable | Recurrence/Total | | HR (95%CI) | *p-*value |
| --- | --- | --- | --- | --- |
| NFS risk categories with 206(59.7) paroxysmal AF patients | | | | |
| Low | | 13/73(17.8) | Reference |  |
| Intermediate | | 40/112(35.7) | 2.38(1.27~4.45) | 0.007 |
| High | | 11/21(52.4) | 4.15(1.86~9.27) | 0.001 |
| NFS as a continuous variable in paroxysmal AF | | | 1.43(1.15~1.78) | 0.001 |
| FIB-4 risk categories with 206(59.7) paroxysmal AF patients | | | |  |
| Low | | 15/83(18.1) | Reference |  |
| Intermediate | | 36/98(36.7) | 2.34(1.28~4.28) | 0.006 |
| High | | 13/25(52.0) | 4.08(1.94~8.59) | <0.001 |
| FIB-4 as a continuous variable in paroxysmal AF | | | 1.28(1.14~1.44) | <0.001 |
| NFS risk categories with 139(40.3) persistent AF patients | | | |  |
| Low | | 6/20(30.0) | Reference |  |
| Intermediate | | 47/94(50.0) | 1.59(0.71~3.56) | 0.256 |
| High | | 17/25(68.0) | 2.79(1.19~6.50) | 0.018 |
| NFS as a continuous variable in persistent AF | | | 1.26(1.01~1.56) | 0.037 |
| FIB-4 risk categories with 139(40.3) persistent AF patients | | | |  |
| Low | | 7/20(35.0) | Reference |  |
| Intermediate | | 40/85(47.1) | 2.05(0.88~4.80) | 0.098 |
| High | | 23/34(67.6) | 3.58(1.41~9.09) | 0.007 |
| FIB-4 as a continuous variable in persistent AF | | | 1.33(0.93~1.90) | 0.117 |

Abbreviations: AF, atrial fibrillation; FIB-4, fibrosis-4; NFS, nonalcoholic fatty liver disease fibrosis score; HR, hazard ratio; CI, confidence interval.
